# Greater Omentum: Multifaceted Interactions in Neurological Recovery and Disease Progression

**DOI:** 10.14336/AD.2024.0213

**Published:** 2024-02-19

**Authors:** Xiang Li, Yuchuan Ding, Yazeed W. Haddad, Xiaokun Geng

**Affiliations:** ^1^Beijing Luhe Institute of Neuroscience, Capital Medical University, Beijing, China.; ^2^Department of Neurology, Beijing Luhe Hospital, Capital Medical University, Beijing, China.; ^3^Department of Neurosurgery, Wayne State University School of Medicine, Detroit, MI 48201, USA.

**Keywords:** greater omentum, inflammatory, perfusion, neurological disease

## Abstract

The greater omentum, a unique anatomical structure composed of adipocytes, loose connective tissue, and a dense vascular network. Plays a pivotal role beyond its traditional understanding. It houses specialized immunological units known as 'Milky spots,' making it a key player in immune response. Moreover, the omentum’s capacity to enhance tissue perfusion, absorb edema fluid, boost acetylcholine synthesis, and foster neuron repair have rendered it a topic of interest in the context of various diseases, especially neurological disorders. This review provides a comprehensive overview of the intricate anatomy and histology of the greater omentum, casting light on its multifaceted functions and its associations with a spectrum of diseases. With a specific focus on neurological ailments, we delineate the intricate relationship that the omentum shares with other pathologies like stroke and we underly its contribution to serving as a therapeutic agent in neurological disorders. By deciphering the underlying mechanisms and emphasizing areas that demand further investigation. This review aims to spark renewed interest and pave the way for comprehensive studies exploring the greater omentum's potential in neurology and broader medicine overall. Given these diverse interactions that yet remain elusive, we must investigate and understand the nuanced relationship between the greater omentum and pathologies, especially its role in stroke's pathophysiology and therapeutic interventions so as to enhance patient care.

## 1. Introduction

The greater omentum is a fibro-fatty tissue beginning with the greater curvature of the stomach, descending down to the symphysis, and covering the abdominal organs. It has a rich network of blood vessels and lymphatic networks, and immune units called milky spots [1].

**Figure 1.**
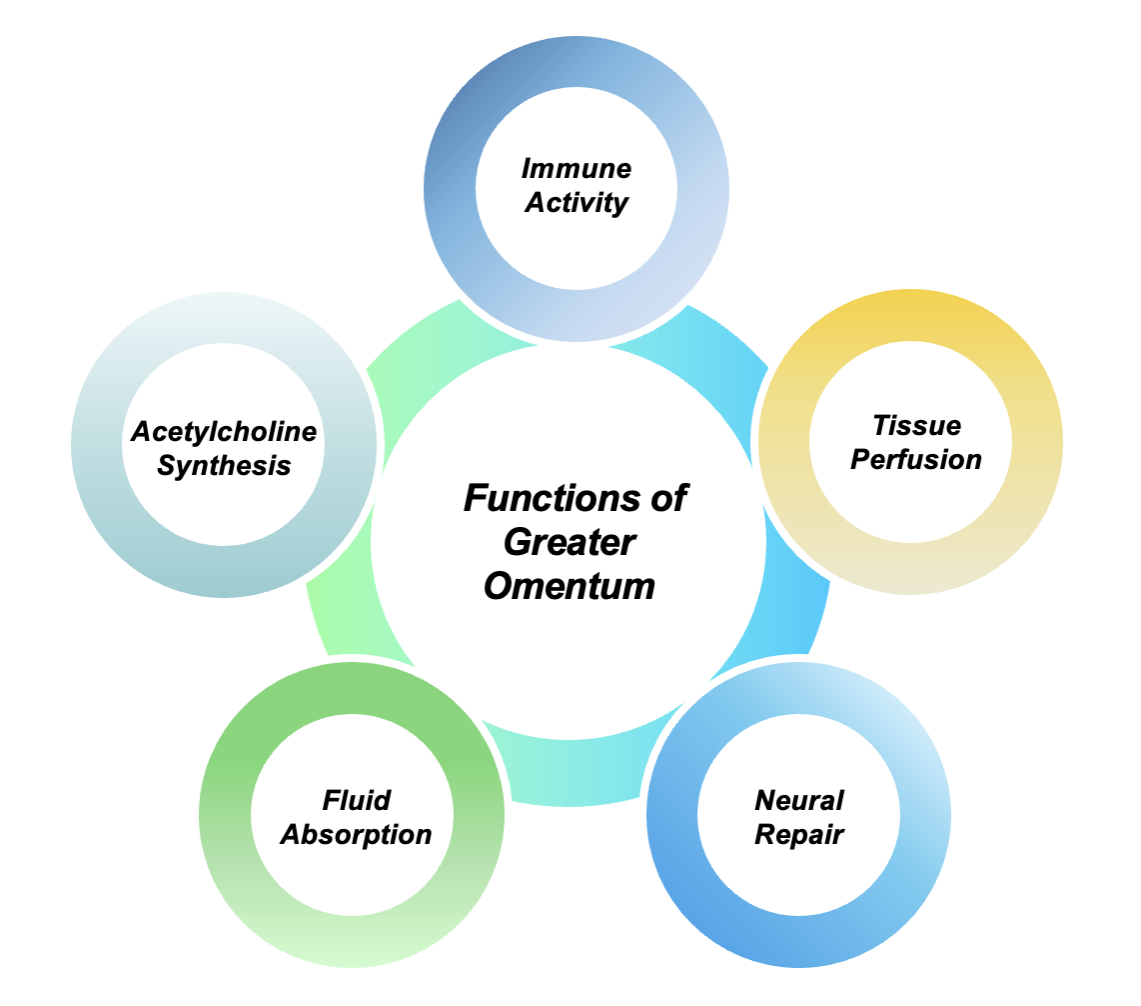
**Multifaceted Functions of the Greater Omentum in Disease**. The greater omentum plays a pivotal role in several physiological processes, crucial to both disease modulation and recovery. It acts as an active immunological organ, contributing to the body's defense mechanisms. By fostering angiogenesis, it aids in optimizing blood flow to affected regions. Its unique structure and composition facilitate efficient uptake and management of edematous fluid. The omentum supports increased production of acetylcholine, a neurotransmitter vital for various neuronal processes. It can bolster neuro-regenerative capabilities, thereby aiding neural restoration following injuries. Together, these functions highlight the greater omentum's significance in pathophysiological contexts and its potential therapeutic applications.

The omentum can provide an additional source of blood vessels and blood flow directly to the tissue where it is placed which makes it a versatile tool to be deployed in special circumstances. It also produces vasoactive substances to promote the synthesis of new blood vessels (angiogenesis) and increase tissue perfusion by increasing the blood vessel density of tissues [2, 3] (Figure 1). Based on the ability of the omentum to increase tissue perfusion, Dr. Goldsmith and colleagues first used the greater omentum for the treatment of stroke in 1979, and patients who accepted the transposition of the greater omentum in brain experienced clinical relief which was one of the first indicators that the greater omentum has a huge scientific and clinical role to play in medicine. This suggests that the greater omentum has a positive impact on the treatment of disease by enhancing tissue perfusion [4]. The findings of Dr. Goldsmith paved the way for further studies that explored the use of the greater omentum in clinical scenarios.

The greater omentum is generally called the ‘‘guardian or policeman of the abdomen’’. Its immune properties make it important in the fight against infections inside the peritoneal cavity [5] (Figure 1). Studies have shown that there are some areas which have significant inflammatory cells and are rich in blood vessels beneath the omentum's mesothelial cell layer between the adipocytes. These areas are commonly known as the milky spots [6]. They are immune units in the omentum and consist of macrophages, T lymphocytes, B lymphocytes, and a small number of other immune cells [7]. When inflammation occurs, inflammatory cells can quickly intercalate the peritoneal cavity through milky spots to inhibit the spread of infection. However, this reaction is not entirely beneficial, and there may be a risk of harm. Peripheral inflammatory cells can also cause autoimmune diseases and organ damage while participating in immune response [8]. As an immune organ rich in inflammatory cells, the omentum may also have this immune injury effect. The inflammatory cells it contains and the inflammatory factors it produces also contribute to the migration of abdominal tumors [9]. This must be considered if the omentum is to be used for clinical purposes. Therefore, a nuanced understanding of the connection between diseases and omental immune function is imperative.

With the increased understanding of the of the omentum and its functions of absorbing edema, synthesizing acetylcholine and promoting neuronal repair. Such examples point to the greater omentum capacity to play an active role in the treatment of diseases and the development of therapeutic techniques that are based on functions that the greater omentum is capable of [10-12] (Fig. 1).

Although the positive role of the omentum in the treatment of diseases has been recognized, whether it has a negative effect on some diseases, including stroke, through immune function and other functions is unclear. At present, the research on greater omentum includes animal studies and clinical studies. While animal studies lay the foundation for the feasibility of greater omental transposition, and the current conclusions on the function of greater omentum are mainly from clinical studies. However, the number of current clinical studies is limited, and more robust powerful clinical evidence to prove the omental function is crucial in the future. The purpose of this review is to clarify the relationship between the greater omentum and diseases by introducing various functions of the greater omentum, so as to promote further research on the relationship between the greater omentum and neurological diseases such as stroke.

## 2. Gross Anatomy of Greater Omentum

The greater omentum is a fibro-fatty tissue, hanging in the abdominal cavity. It starts at the stomach's greater curvature, attaches the transverse colon, and passes in front of it. It then descends to the anterior pelvic junction in front of the abdominal organs. The dimensions and surface area of the omentum can vary within a certain range. It can vary from 14cm to 36cm in length and from 20cm to 46cm in width, and its surface area can vary from 300cm^2^ to 500cm^2^ [1]. The mesothelial cell layer of the omentum provides a protective non-adherent surface, forming a near frictionless interface with the peritoneum and visceral serosa. This may be one of the main roles that the omentum serves in the abdominal cavity [13]. The left gastroepiploic artery and the right gastroepiploic artery provide a rich blood supply to the greater omentum. The left gastroepiploic artery comes from the splenic artery and the right comes from the gastroduodenal artery. These arteries connect to each other and form an arcade along the greater curvature of the stomach, and together with their accompanying veins form a rich network of blood vessels in the omentum [1]. The lymphatic vessels of the omentum originate from immune units in the omentum called milk plaques. Like the arteries and veins in the omentum, they are interconnected to form a complex network structure. These vessels travel through the fatty tissue of the omentum and enter the subpyloric and splenic lymph nodes respectively [1].

## 3. Histological Feature of Greater Omentum

The greater omental tissue consists of fat-rich and translucent regions of similar architecture. These regions are constructed from up of two single-layer mesothelial sheets sandwiched by submesothelial connective tissue. There are multiple fenestrations up to 225μm in diameter in this bilayer [13]. Cells and some small molecules can move between the anterior and posterior lobes of the larger omentum and the peritoneal cavity attributable to these fenestrations. There are many areas in the adipose-rich regions of the greater omentum called milky spots, which are densely packed with vital inflammatory cells and blood vessels. The area of milky spots is about 0.3 to 3.5 mm^2^ and its density decreases to 2 per cm^2^ with age [6]. They are usually arranged in a circular or irregular shape around the blood vessels in the greater omentum [14]. Milky spots cover mesenchymal fenestrations in the adipose-rich area, which act as entry points for inflammatory cells to enter the peritoneal cavity following infection [15]. A special type of postcapillary venules called high endothelial venules is important in the milky spots, which are critical for lymphocyte transport [16]. However, there are also avascular or so-called secondary milky spots [17]. Milky spots contain sympathetic nerve fibers, which are usually found around small blood vessels and occasionally between immune cells. By releasing neurotransmitters, these neurons may modulate immune cell activity and add to the greater omentum's clinically significant characteristics during the treatment of abdominal infection [18].

## 4. Immunologic Function of Greater Omentum

The immunological units of the greater omentum are referred to as milky spots, which are composed of macrophages, B and T lymphocytes, and a few other immune cells [7]. There are fenestrations between the mesothelial cells covering milky spots, and the associated basal lamina in the mesothelial cell layer is absent [19]. This histological structure allows inflammatory cells in milky spots to quickly enter the peritoneal cavity to participate in the immune response during inflammation. High endothelial venules in milky spots recruit circulating neutrophils and act as outlets for rapid neutrophils entry into milky spots and release into the peritoneal cavity during early inflammation [20]. Through high endothelial venules, other inflammatory cells, such as B and T lymphocytes, can also reach the peritoneal cavity [6]. Like the reaction that happens in blood vessels during inflammation, there is a marked rise in the size and quantity of milky spots when infection occurs in the peritoneal cavity. The explanation for the rise in milky spot size is because infection stimulation leads to an enormous number of neutrophils into the milky spots, which is followed by an increase in lymphocytes and macrophages [21]. Furthermore, for a period of time after infection, as the number of inflammatory cells decreases, the reticular connective tissue network forms in the milky spots, which expands from the center of the milky spots, thereby transferring inflammatory cells to the edge of the milky spots [21]. Macrophages and B1 cells are the main immune cells in milky spots [22]. Abdominal inflammation can cause macrophages in the peritoneal cavity to quickly migrate to the greater omentum a process known as the macrophage disappearance reaction [23, 24]. The macrophages concentrate around foreign bodies, showing phagocytic phenotype [21]. B1 cells are a distinct subset of B cells with the capacity to generate natural antibodies. The chemokine CXCL13 localizes them to the omentum, where they support the adaptive immune response [25, 26].

## 5.Greater Omentum and Tissue Perfusion

The greater omentum has a rich blood supply network, and it can provide an additional source of blood vessels to any area of the body through transplantation. Blood flow from the omentum can be deeply perfused to the underlying location upon which it had been placed [2]. This property increases blood flow to the tissue where the greater omentum is placed.

Furthermore, a study has shown that in the absence of ischemia in the tissue where the greater omentum is placed, the covered omentum also generates protective collateral circulation to the tissue to improve perfusion. This is due to the presence of substances in the omentum that promote angiogenesis [27]. The angiogenic function of the greater omentum is mainly related to vascular endothelial growth factor (VEGF). VEGF is a heparin-binding glycoprotein, which has a strong role in promoting endothelial cell specific mitosis and angiogenesis [28, 29]. VEGF is abundant in adipose tissue, and the greater omentum contains and secretes the highest level of VEGF [30]. This ability to synthesize VEGF is attributed to CD34-expressing stem cell population in the omentum [31]. Inflammation and hypoxia can induce the expression of VEGF in omental cells, and then induce angiogenesis to reduce the damage caused by inflammation and hypoxia [2, 30].

In addition to VEGF, fibroblast growth factor (FGF) also promotes angiogenesis [32, 33]. Similar to VEGF protein, FGF is also synthesized by CD34-expressing stem cells in the greater omentum [31]. Similarly, neurotrophic factors can also promote vascular regeneration of the nervous system and other systems after ischemic insult [34]. Currently, five different neurotrophic factors have been described, including brain-derived neurotrophic factor (BDNF), nerve growth factor (NGF), and neurotrophic factor (NT) 3,4, and 6. Almost all neurotrophic factors can be significantly expressed in the greater omentum [35]. This suggests that neurotrophic factors may be involved in the ability of the greater omentum to promote angiogenesis.

## 6.Other Functions of The Greater Omentum

In addition to its immune function and its role in enhancing tissue perfusion, the omentum has other functions associated with diseases.

### 6.1. Absorbing Edema Fluid

The omentum contains a rich vascular network and lymphatic network, which has aroused interest in whether the omentum can absorb edema after displacement [1]. One study showed that the continuity of lymphatic flow in the legs of experimental animals after greater omental displacement was significantly restored compared to untreated control animals [12]. Moreover, a clinical trial demonstrates that the symptoms of leg edema in a patient with leg lymphedema were significantly improved after transposition of the greater omentum [2]. This suggests that edematous fluid from the body's periphery may be absorbed by the omentum. Furthermore, research has demonstrated that the greater omentum's surface area, along with the quantity of blood arteries and lymphatics, enable the omentum to absorb edema fluid. The omentum displaced to the spinal cord can transport up to 30% of all cerebrospinal fluid volume through the omental channel [36]. The omentum absorbs edema fluid mainly through capillaries and lymphatics. In steady state, most of the fluid (60%-80%) flowing into the greater omental tissue is absorbed by capillaries, while a small part (20%-40%) is absorbed by lymphatic vessels [37].

### 6.2. Increasing Acetylcholine Synthesis

The omentum contains a variety of neurotransmitters, including dopamine, norepinephrine, epinephrine, and choline acetyltransferase (ChAT), a marker for acetylcholine (Ach). Ach is an important excitatory neurotransmitter involved in higher behaviors such as learning and memory. ChAT is an enzyme that can synthesize choline and acetyl-CoA into acetylcholine, which is one of the main components of cholinergic neurotransmission [38]. Other studies have shown that, due to the presence of ChAT activity in retinal tissue, translocation of ChAT to the back of the brain may enhance cholinergic neurotransmission in the brain [10]. However, the presence of highly active cholinesterase in the blood indicates that the retinal membrane displaced to the brain cannot directly increase Ach levels in the brain by transporting Ach synthesized in the retinal membrane to the brain through newly established vascular connections. Currently, it has been suggested that oral choline administration in conjunction with transposition of the greater omentum may increase acetylcholine synthesis in remaining functional neurons in the brain [38]. The mechanism by which the greater omentum enhances cholinergic neurotransmission in the brain still needs further study.

### 6.3. Promoting Neuron Repair

It was reported that the greater omentum can promote neuron repair through a variety of mechanisms. Laminin is a kind of extracellular matrix molecules, which can participate in the spontaneous axon adhesion, migration and regeneration of central nervous system and peripheral nervous system by binding with integrins [39]. Studies have shown that the basal layer of the omentum is rich in laminin, suggesting that the displaced omentum can promote the repair and regeneration of the nervous system [11]. Furthermore, studies have shown that VEGF and FGF produced by the omentum not only promote the function of angiogenesis, but also support the migration of neurons. They can promote the migration of neural stem cells to the injured site, so that the damaged site neurons can be repaired [40]. In addition, NGF and BDNF have the function of promoting axon growth and ultimately neuron repair [41-43]. This means that the omentum, which is capable of producing neurotrophic factors, may play a role in pathology affecting the nervous system.

**Figure 2.**
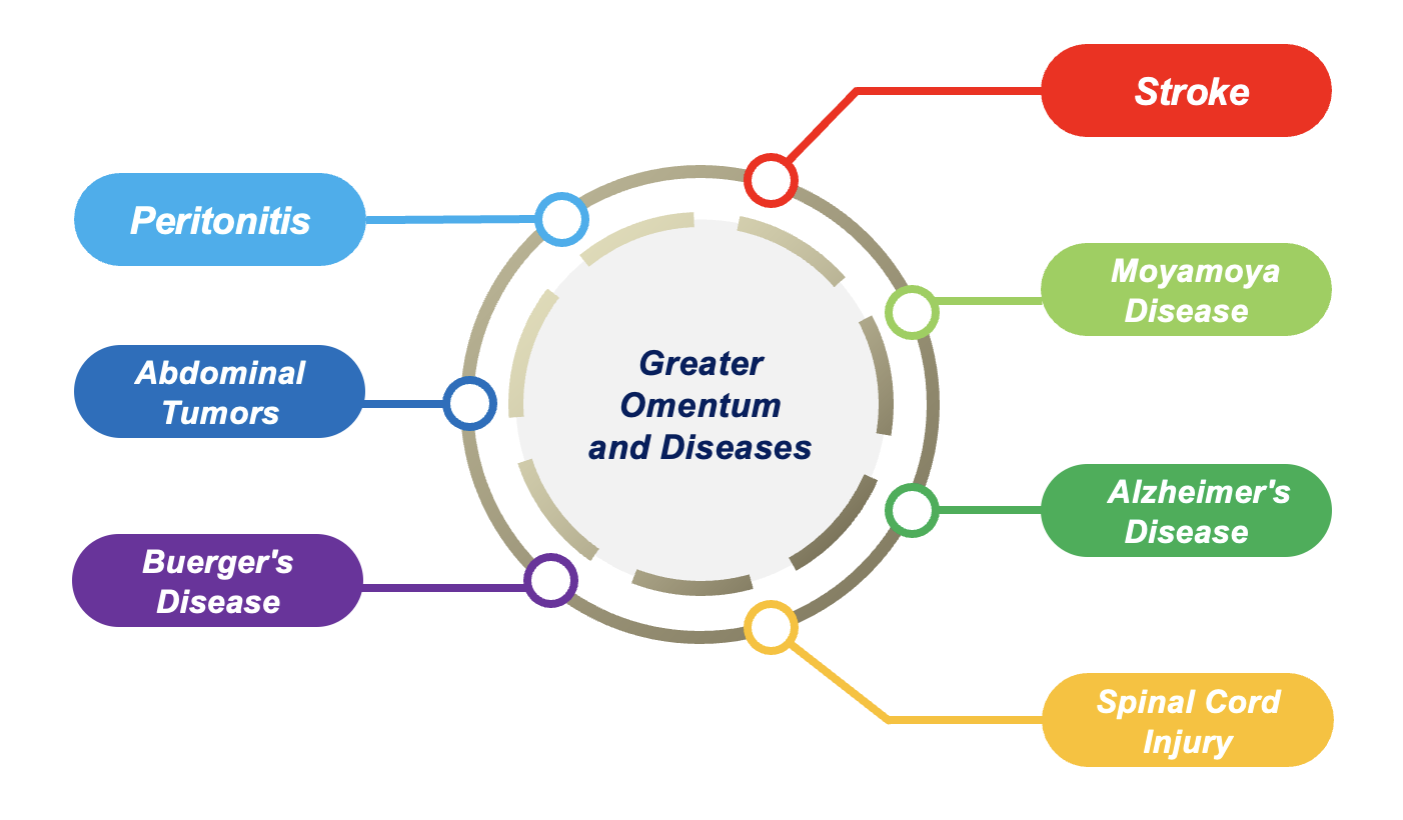
**Multifaceted Impact of the Greater Omentum on Various Diseases**. The greater omentum has profound associations with a range of diseases, both as a therapeutic agent and as a factor contributing to pathology. In Neurological Affiliations, Research suggests the omentum may play a beneficial role in post-stroke recovery, potentially due to its angiogenic and anti-inflammatory properties. Its functions such as increasing tissue perfusion and promoting acetylcholine synthesis make it have a positive impact on the treatment of AD. Moreover, the omentum has been indicated to support revascularization efforts in Moyamoya patients. Preliminary studies hint at the omentum's potential in modulating the disease's progression or its symptomatology. The angiogenic and neuroprotective capabilities of the omentum might aid in the repair and regeneration processes following spinal injuries. In non-neurological diseases, the immune function of the omentum and its ability to increase tissue perfusion generally contribute positively to the body's defense mechanisms as well as to the treatment of diseases, for example, the omentum has a beneficial effect on the treatment of Buerger's disease patients by increasing blood flow to the ischemic limb. But in conditions like peritonitis, they can exacerbate organ damage. The omentum can inadvertently promote the movement or displacement of abdominal tumors, potentially complicating clinical scenarios. Given these diverse interactions, there's an imperative to investigate and understand the nuanced relationship between the greater omentum and diseases, especially its role in stroke's pathophysiology and therapeutic avenues.

## 7.The Greater Omentum in Diseases

The greater omentum is a fiber-adipose tissue. Its ability to increase tissue blood flow and recruit and produce Inflammatory cells has linked it to neurological and other systemic diseases. Studies have shown that greater omentum transplantation has a positive effect on the treatment and prognosis of neurological diseases including stroke, moyamoya disease, Alzheimer's disease and spinal cord injury. In other systemic diseases, the omentum inhibits the occurrence and development of abdominal inflammation through its immune function, but inflammatory cells involved in the immune response can cause damage to other organs. Its immune activity also promotes the metastasis of abdominal tumor to omentum. In addition, transplantation of omentum has a positive effect on the treatment and prognosis of thromboangiitis obliterans (Fig. 2).

### 7.1. Application of Greater Omentum in Treatment

At present, the greater omentum has been transferred to the brain and spinal cord to treat neurological diseases, which benefits from its unique anatomical structure and functional characteristics. The omental membrane has a rich network of blood vessels, which allows the omental pedicled flap to be flexibly shaped according to the blood supply of the displaced site [44]. Moreover, it is able to absorb the edema fluid from the surrounding tissue after the omentum transfer, reducing the production of scar tissue, which greatly improves the success rate of omentum transfer surgery [45]. Furthermore, the immune function of omentum makes it capable of tissue regeneration, revascularization and inflammation control [44]. The ability of omentum to promote cholinergic activity and produce neurotrophic factors further suggests that it has great potential as an effective clinical intervention [10, 11].

In order to verify the feasibility of transplanting omentum into the brain, experiments were conducted with dogs and monkeys as experimental animals. In the study, which used dogs as experimental animals, the omentum was transferred to the brain through subcutaneous tunnels in the chest and neck, and none of the dogs experienced any adverse reactions as a result of the surgery over a nine-month period. Subsequent studies have shown that extensive vascular connections in the omentum can enter the dog's brain to increase brain perfusion [46]. This suggests that transposition of the omentum to the brain is anatomically feasible for dogs. Since the ratio of annular gray matter to white matter in dogs is similar to that in human brains, and the cerebral arterial circulatory system in dogs is similar to that in humans, greater omental transposition may be feasible in humans. However, as compared with humans, the middle cerebral artery in dogs receives basal cranial circulation from the anterior cerebral artery and the posterior cerebral artery. In addition, the maxillary carotid artery in dogs also has significant collateral circulation. In addition, studies using monkeys as experimental animals show the positive result [27]. Taken together, additional clinical studies are needed to further determine whether transposition of the greater omentum is feasible in humans and whether transposition of the greater omentum can play corresponding roles in human brain [47].

In omental transposition, the common approach is to remove the omentum from the peritoneal cavity through an incision in the abdomen and to reach the brain or injured spinal cord through a subcutaneous tunnel in the chest and neck. The omentum is then sutured to the dura and fixed in place [48]. This kind of omental transposition has been used in stroke, spinal cord injury and other nervous system diseases, and the symptoms of patients have been relieved to varying degrees without obvious surgical side effects [45]. In addition, studies have reported another method of omental displacement in spinal cord injury, namely the use of free omental grafts instead of omental pedicled flaps. After the free epiploid graft is placed in the injured spinal cord, the gastroepiploid artery and vein are sutured to the neck vessels. However, this method of transplantation is not only technically difficult, but also may make the transplanted omental tissue lose its function of absorbing edematous fluid [49]. Therefore, it is necessary to further explore more effective omental displacement methods.

While the greater omentum has shown promise in facilitating neurological recovery due to its rich vascular supply and lymphatic tissue, which can aid in neuroprotection and neuroregeneration, there are specific concerns that need to be addressed. As a surgical procedure, some complications would inevitably occur after greater omental transplantation. Studies have shown that the most common complication after greater omentum transplantation is abdominal wall infection, followed by fascial separation. Furthermore, symptomatic abdominal hernias, as the third most common complication, occur only in surgery where a vascularized greater omentum is used for transplantation. In addition, there are unusual complications such as pyloric obstruction, intestinal obstruction and delayed splenic rupture [50, 51]. Side effects caused by the functional properties of the omentum should also be considered. As an immune organ, the immunomodulatory properties of omentum are beneficial to promoting recovery of the nervous system [52]. But in an environment where the central nervous system is weak, there may be a risk of an excessive immune response. In addition, omental transplantation may cause rejection and changes in nerve function. Further investigation of these potential side effects is needed in the future.

### 7.2. Neurological Disease

### 7.2.1. Stroke

Stroke is a cerebrovascular disease in which the brain's blood arteries suddenly burst or become blocked, preventing blood from reaching the brain and causing damage to its tissue. These are known as ischemic or hemorrhagic strokes respectively. Most strokes are ischemic strokes [53-55]. The sequela due to cerebral infarction, cause severe brain and neuronal damage, including brain tissue damage and structural damage, as well as neuronal death [56-58]. Most of the initial clinical deficits in stroke patients come from a low perfusion and electrically inactive brain region known as the ischemic penumbra, which gradually converts to irreversible damaged tissue over time. However, this area can be effectively saved by restoring brain tissue perfusion [59]. The ultimate therapeutic goal of ischemic stroke is to reduce neuronal damage by relieving arterial occlusion and restoring cerebral perfusion [60, 61]. Study has indicated that following stroke, carotid endarterectomy can improve cerebral blood flow and facilitate blood flow to non-ischemic brain regions. However, ischemic regions of the brain where blood arteries are clogged, are difficult for blood to reach. There is currently no medication or surgical technique that can permit a significant volume of blood to flow into the brain's ischemic location through the extracerebral artery for a prolonged length of time [62, 63].

**Table 1:** The function of greater omental transposition in the treatment of disease is included in the manuscript. Because of its unique anatomical structure and physiological functions, the greater omentum has been used in the treatment of diseases. In neurological diseases, omental transposition can play a positive role in the treatment of stroke, moyamoya disease, Alzheimer’s disease and spinal cord injury. In addition, the omentum can also be used in the treatment of non-neurological diseases, such as Berger's disease, which is closely related to its ability to increase tissue perfusion.

Disease	Function of the greater omental transposition	Current status
**Stroke**	Increase perfusion of ischemic brain tissue	Case Reports [4,65,66]
**MoyaMoya Disease**	Promote the synthesis of new blood vessels to restore cerebral blood supply	Case Reports [50,73-76]
		Observational Study [77]
**Alzheimer's Disease**	Restore cerebral blood supply to increase blood flow to brain tissue	Case Reports [88]
	Enhance cholinergic activity	Case Reports [10]
	Production of growth factors that promote neuronal repair	Clinical Trial [89]
**Spinal Cord Injury**	Increase perfusion to the injured spinal cord	Case Reports [93]
**Buerger's Disease**	Provide additional blood perfusion to ischemic tissue	Case Reports [104,105]

The greater omentum can increase perfusion of the tissue in which it is placed by providing additional blood vessels and blood flow and promoting the synthesis of new blood vessels. The ability has stimulated interest in its role in stroke treatment [2]. Goldsmith HS and colleagues first used the greater omentum in 1979 in the treatment of two stroke patients with expressive language disorders, and measured improvements in brain function by objective progress in the patients' ability to communicate. After treatment, the patient's speech content, fluency and spontaneity were significantly improved [4]. It is suggested that the greater omentum has a positive effect on the treatment of cerebral apoplexy. Another clinical study showed that a stroke patient treated with transposition of the greater omentum had significant improvement in expressive aphasia, right hemiplegia, and inability to read over a long period of time. This is due to the greater omentum's ability to increase tissue perfusion. After the greater omentum is transferred to the brain, the additional cerebral blood flow provided by it can have a positive impact on the cells in the ischemic penumbra around the infarction site. The greater omentum also can increase collateral circulation of ischemic brain tissue by producing vascular growth factor, thereby increasing the blood flow of brain tissue [64, 65]. In this study, the ability to read of stroke patients gradually began to recover after the greater omental transposition, which explains the possibly causal relationship between the greater omental transposition and stroke, which the greater omental transposition improves the nerve defect symptoms of stroke patients by increasing the perfusion of brain [65]. Similarly, in a subsequent study, the omentum improved neurological function in three patients with cerebellar or brainstem ischemia through its ability to increase tissue perfusion. This again confirms the causal relationship of greater omental transposition and stroke [66](Table 1). While increasing brain perfusion is not the only mechanism in which greater omentum plays an active role in stroke treatment, the immune ability and ability to produce neurotrophic factors may be the key factors as well. Studies have proved that peripheral macrophages have neuroprotective effects and can promote the repair of the central nervous system after the acute phase of stroke [67]. Owe to the immune capacity of the omentum, the displaced omentum may exhibit nerve restoration through this immunomodulatory pathway during stroke. Moreover, VEGF, FGF and neurotrophic factors produced by the omentum also have the function of promoting neuronal repair after injury, and thus may have a positive impact on nerve function impairment caused by neuronal ischemic death during stroke [68]. Although studies have demonstrated the positive role of omentum in the treatment of stroke, there are still shortcomings in the studies. For example, the sample size of these studies was limited, and no conclusions can be drawn that are generally applicable to the entire population. Moreover, in stroke treatment, researchers only used pedicled greater omental transposition, and did not study other methods of greater omental treatment. In addition, the study lacked long-term follow-up data, and the long-term effects and stability of omentum intervention were unclear. And there is a lack of detailed mechanism. These deficiencies suggest that further studies on the relationship between the greater omentum and stroke are needed.

### 7.2.2. Moyamoya Disease

The unusual growth of the vascular network and steno occlusive alterations in the circle of Willis are the hallmarks of moyamoya disease, an uncommon cerebrovascular illness [69]. The clinical features of patients with moyamoya disease are usually ischemic or hemorrhagic presentation, while a small number of patients have both ischemic and hemorrhagic symptoms [70]. The most reasonable way to treat this disease is to construct vascular anastomosis channels to increase perfusion to ischemic brain tissue [71, 72].

The greater omentum has the function of providing additional blood vessels, blood flow and of synthesizing angiogenic factors to generate blood vessels. This suggests that greater omentum transposition is a promising method for the treatment of moyamoya disease. Karasawa and colleagues first performed omental transposition in a quadriplegic and blind moyamoya patients in 1978. After surgery, the cerebral vessels in the patient's cerebral cortex began to rebuild, and the patient was gradually able to walk with assistance [73]. By observing the treatment and prognosis of patients with moyamoya disease with greater omental transposition, this study demonstrated the causal relationship between greater omentum and the improvement of neurologic deficits caused by moyamoya disease. Furthermore, compared with patients treated with superficial temporal artery-middle cerebral artery anastomosis for moyamoya disease, patients treated with greater omentum have increased perfusion in the anterior and posterior cerebral artery regions and improved neurological symptoms caused by ischemia in this region, so greater omental transposition is often used as the preferred treatment for most patients with moyamoya disease. This study strongly demonstrated that the transposition of the greater omentum to the brain can improve the symptoms of neurological impairment caused by cerebral ischemia [74]. Another study showed that in 30 pediatric patients undergoing greater omental transposition surgery, the neurological function of almost all patients improved after surgery, and the recovery rate of patients was correlated with the number of newly formed collateral circulation vessels [75]. A series of subsequent studies suggested that ischemic symptoms could be effectively reduced in almost all patients treated by omental transposition [51, 76-78]. This is attributed to the function of the omentum by providing additional sources of blood vessels and synthesizing VEGF to promote the formation of new blood vessels and increase cerebral perfusion (Table 1). In addition, similar to ischemic stroke, the greater omentum may repair neurons damaged in moyamoya disease, owing to neurotrophic factors produced by the omentum and its immune function [68]. Although this study has demonstrated the therapeutic effect of omentum on moyamoya disease, the small sample size and short follow-up time emphasizes the necessity of future study with large samples and long-term follow-up.

### 7.2.3. Alzheimer's Disease (AD)

AD refers to specific episodes and processes of age-related cognitive and functional decline, characterized by the clinical manifestations of generalized dementia, and ultimately leading to death [79-81]. Decreased cerebral blood flow is a normal physiological phenomenon that changes with age. Diabetes, hypertension and other adverse factors in daily life can also lead to reduced cerebral blood perfusion [82]. However, in almost all cases of AD, malformed brain capillaries develop into abnormally shaped capillaries, which also lead to a decrease in cerebral blood flow [83]. The movement of oxygen, glucose, and other micronutrients across the blood-brain barrier as well as the brain's ability to expel catabolites are all impeded by a reduction in blood flow to the brain. Eventually, it will adversely affect the activity of neurons, especially ischemia-sensitive cells [2, 84]. This suggests that improving cerebral blood flow can play a positive role in the treatment of AD patients. Besides, studies have shown that AD can cause damage to the cholinergic system in the brain, with severe neurodegeneration, reduced cholinergic neurons, and Ach deficiency. Therefore, restoring the cholinergic system after injury is also one of the clinical therapies to treat AD [85-87].

The ability of the greater omentum to increase tissue perfusion and enhance cholinergic neurotransmission raises the possibility of what effect the transposition of the omentum to the brain might have on AD. The first greater omentum transfer operation was performed in AD patients in 1993. After the operation, the nerve function of the patient gradually improved, and neuroradiological examination showed that the perfusion of the patient's brain tissue increased significantly, especially the brain tissue located below the omentum placement [88]. This is due to the ability of the displaced omentum to provide additional vascular and blood flow functions. Increased perfusion of brain tissue brings more glucose, oxygen, and micronutrients to the brain, allowing ischemic neurons to recover, thereby relieving symptoms in patients. In addition to the function of increasing blood perfusion of brain tissue, the positive role of omentum in the treatment of AD may also be related to its ability to increase the synthesis of acetylcholine and produce neurotrophic factors. Another study showed that patients with AD who were treated with a greater omentum transplant experienced a significant improvement in their clinical symptoms and were significantly more likely to experience clinical improvement than patients with AD who were treated with cholinesterase inhibitors therapy (CHEI) [89]. In addition to the restoration of cerebral perfusion, the growth factor produced by the omentum to promote the repair of injured nerves is also one of the reasons for the improvement of clinical symptoms. Studies have shown that because of the function of the greater omentum to synthesize acetylcholine, transposition of the omentum into brain tissue may enhance cholinergic neurotransmission in brain tissue. The greater omentum may relieve clinical symptoms caused by damage to the cholinergic system through this function [10]. Moreover, the ability of the omentum to promote neuronal repair in neuroactivity-deficient areas has been noted, which may be related to neurotrophic factors produced by the omentum [35] (Table 1). Although there is a variety of mechanisms by which omentum can benefit AD patients, there are problems such as limited sample size, single clinical intervention methods of omentum, lack of long-term follow-up, and insufficient explanation of other omentum related mechanisms. Therefore, further studies on omentum treatment of AD are of great importance.

### 7.2.4. Spinal Cord Injury (SCI)

SCI is a devastating condition that can cause severe sensory and motor dysfunction below the injury plane, eventually leading to severe disability and even death. Myelin and neuronal axons sustain primary damage from SCI, which is followed by secondary damage resulting from the release of cytotoxic factors and other processes [90, 91]. The treatment strategies of SCI are divided into neuroprotection and nerve regeneration. Neuroprotection aims to reduce inflammation, while neuroregeneration aims to promote the repair of neurons and the regeneration of axons. However, there is currently no viable treatment for SCI because of the limited capacity for spinal cord regeneration and the presence of inhibitory chemicals at the damage site [11].

The greater omentum has a rich network of blood vessels and immune units called milky spots, which can increase tissue perfusion, absorb edema, and promote immune response. This suggests that the greater omental displacement may be an effective treatment for SCI. Studies have shown that transposition of the omentum into the spinal cord can promote revascularization after SCI, owe to the fact that the omentum directly provides additional vascular sources for the spinal cord at the site of injury and secretes VEGF to promote the formation of neovascularization [92]. A clinical study has shown that omentum transplantation can promote the recovery of damaged spinal cord function, which is due to its ability to provide rich blood to the damaged spinal cord and promote the synthesis of capillaries [93](Table 1).

Furthermore, the greater omentum can produce immune cells and anti-inflammatory factors which fight inflammation [6]. Revascularization and the release of anti-inflammatory cells and factors can reduce the inflammatory response around the site of spinal cord injury, indicating that the omentum has a neuroprotective function in SCI [11]. Moreover, the greater omentum displacement can also have a positive effect on SCI by absorbing vasogenic edema caused by injury to the spinal cord. It can reduce the fibrinogens produced after SCI by absorbing edema, thus limiting the formation of scar at the injury site, which has a positive effect on the repair of neurons [11, 92]. In addition, the characteristic of the greater omentum to promote nerve tissue regeneration at the injured site are essential for the treatment of SCI. This characteristic is probably related to laminin in the stroma of the omentum, its production of vasoactive growth factor and neurotrophic factor [11, 40, 42]. With the in-depth understanding of the interaction mechanism between the greater omentum and the injured spinal cord, it is necessary to further explore the potential of the greater omentum in the treatment of SCI.

### 7.3. Non-neurological Disease

### 7.3.1. Peritonitis

Peritonitis can be caused by a variety of risk factors, such as perforation of abdominal organs, gastrointestinal anastomotic fistula, penetrating infection or cancer [94]. The main clinical manifestations are abdominal tenderness, abdominal muscle tension and rebound pain. During peritonitis, the peritoneal cavity can become infected as a result of contamination, initiating an inflammatory response to suppress the infection. If the infection cannot be controlled or eradicated, the intra-abdominal sepsis it causes can lead to systemic injury with high mortality and global organ dysfunction [95].

As an intraperitoneal organ, the greater omentum has the function of reducing peritoneal inflammation and promoting wound recovery, and is often referred to as the abdominal police [5]. Some studies have shown that omentectomy has adverse effects on both experimental animals and humans [96, 97]. This proves that the greater omentum plays an active role in controlling abdominal infection. However, studies have shown that although neutrophils have a defensive role against infection during peritonitis, they can also cause damage to distal organs [8]. Milky spots in the omentum are the main ways for neutrophils to enter the abdominal cavity during peritonitis, and they also play a role in recruiting neutrophils in circulation. [20, 95]. It is suggested that omentum not only controls infection by participating in immune response in peritonitis, but also may have a negative effect on distal organ injury. In view of the fact that the greater omentum may cause immune damage to other organs while playing an anti-infective role, it is necessary to further explore the relationship between the immune function of the greater omentum and diseases.

### 7.3.2. Abdominal Tumors

Malignant cells of primary abdominal tumors, such as ovarian cancer, colon cancer and gastric cancer, usually metastasized to the peritoneal cavity, among which the greater omentum is the most common tissue for tumor metastasis [98]. Studies have shown that although macrophages in the milky spots of the greater omentum have toxic effects on tumor cells and can reduce the number of tumor cells in the milky spots, macrophages cannot eliminate all tumor cells in the omentum except the milky spots, and these tumor cells will gradually grow [99].

Tumor cells which metastasized to the abdominal cavity preferentially attach to the milky spots of the omentum, and this attachment occurs very rapidly [9]. Studies have shown that the selective attraction of tumor cells expressing CCR4 to macrophages expressing CCL22 in milk plaques is responsible for this occurrence [100]. Moreover, other studies have shown that the release of a chromatin network known as the neutrophil extracellular trap (NET) by neutrophils in the abdominal cavity can be triggered by inflammatory stimuli coming from ovarian tumors. This NET attaches itself to ovarian cancer cells and encourages their spread to the larger omentum [101]. Moreover, CD163+ Tim4+ macrophages in the omentum play an important role in the metastasis and diffusion of ovarian cancer in the omentum [102]. However, in addition to chemokine-dependent migration to the omentum and milky spots, tumor cells may also passively accumulate in the omentum due to peritoneal fluid flowing through the omentum and milky spots from the peritoneal cavity [98]. In view of the fact that immune cells produced by the greater omentum can promote the migration of abdominal tumors, it is necessary to further explore the relationship between the greater omentum and other tumor diseases.

### 7.3.3. Buerger's Disease

Angiitis obliterans, also known as buerger's disease, is a segmental thrombotic obliterans disease that often involves small and medium blood vessels. Its main clinical manifestations are intermittent claudication, resting pain and paresthesia, with body ulceration and necrosis in severe cases [103].

The greater omentum is rich in vascular networks and has the ability to increase tissue perfusion. Therefore, transposition of the greater omentum is considered to be an effective treatment for buerger's disease. A clinical study shows that four patients with buerger's disease who presented with severe lower limb ischemia experienced significant improvement in clinical symptoms, including pain relief and healing of distal limb ulcers. In addition, postoperative angiography showed increased collateral vessels, suggesting that the greater omentum plays a positive role in the treatment of buerger's disease by providing additional blood vessels for ischemic muscle and generating angiogenesis through the production of vascular growth factor [104]. Another study showed that three patients have improved clinical function after omental transposition, including pain alleviation and increased walking distance, based on the function of omental transfer to promote blood vessel synthesis and increase tissue perfusion [105](Table 1).

## 8.Perspectives and Prospective

The greater omentum is a type of fibrous adipose tissue. Its immune function, increased tissue perfusion, absorption of edematous fluid, synthesis of acetylcholine, and promotion of nerve repair make it relevant to a variety of diseases, including neurological disorders. The relationship between the omentum and diseases of various systems has attracted extensive attention. Animal and clinical studies have shown that omentum has a positive impact on the treatment and prognosis of neurological diseases through its unique function. However, in some non-neurological diseases, not only may the omentum have a positive impact on the treatment and prognosis of the disease, but it may also cause damage to certain organs due to its immune effect.

In the context of neurological diseases, the specific role of the omentum deserves further exploration. At present, studies on the relationship between greater omentum and neurological diseases include observational and retrospective studies, and different studies include children, middle-aged people, and the elderly of different genders. Clinical intervention using pedicled omentum transplantation was performed and changes in neurological function were assessed. Although different studies have different methods to evaluate the neurological function in patients, the neurological function has been significantly improved after the intervention of omental transplantation. This further emphasizes the positive role of omentum in the treatment and prognosis of nervous system diseases. Although almost all studies on the relationship between greater omentum and nervous system diseases suggest the positive role of omentum in clinical intervention, the immune properties of the omentum may promote tumor metastasis and possibly cause distal organ damage. Future studies should focus on the immune capacity of omentum to understand its role as an immune organ and its influence on neurological diseases. As we learn more about the relationship between the greater omentum and neurological diseases, we are likely to become more aware of its positive or negative effects in these diseases.
